# Older Veterans With Chronic Pain Who Persist in Group Exercise Show Improved Mobility

**DOI:** 10.1093/geroni/igaa057.2790

**Published:** 2020-12-16

**Authors:** Odessa Addison, Jamie Giffuni, Megan Kelly, Beth Hogans, Leslie Katzel, Miriam Morey

**Affiliations:** 1 VA Maryland Healthcare System, Baltimore, Maryland, United States; 2 Baltimore VA Healthcare System, Baltimore, Maryland, United States; 3 Geriatric Research, Education, and Clinical Center, VA Maryland Health Care System, Baltimore, Maryland, United States; 4 Veterans Health Administration, Durham, North Carolina, United States

## Abstract

Approximately 50% of older adults receiving care at VA are Veterans with chronic pain (V-CP). While physical activity can reduce chronic pain and increase mobility, little is known about group exercise (GE) effects for V-CP. We hypothesized that attrition may limit GE effectiveness. In this study, we retrospectively compared program attrition and participant mobility at three-months of Gerofit GE. At baseline, Older V-CP (N=21) had lower mobility assessment scores (gait speed, 6MW, chair stands, TUG: 1.04 m/s, 443 yd, 11, 9.35s vs. 1.09 m/s, 463 yd, 12, 8.19s respectively) compared to unaffected Veterans. Three-month attrition was higher for older V-CP (54% vs 39%). For those completing three months GE, gains in mobility were similar. We conclude that Veterans with chronic pain are less likely to persist in group exercise but those who persist benefit much like those without pain. Further study is needed to understand successful exercise adherence.

